# Thanatochemistry and the role of hypoxanthine in the post-mortem interval estimation: a systematic literature review

**DOI:** 10.1007/s00414-024-03378-x

**Published:** 2025-02-22

**Authors:** Andrea Nicola Cardinale, Antonio Di Lorenzo, Mara Bellino, Giuseppe Strisciullo, Valentina Mussi, Sara Sablone

**Affiliations:** 1 Section of Legal Medicine, Interdisciplinary Department of Medicine, University of Bari Aldo Moro, Bari Policlinico Hospital, Bari, Italy; 2 Interdisciplinary Department of Medicine, University of Bari Aldo Moro, Bari Policlinico Hospital, Bari, Italy; 3 Toxicology Laboratory, Interdisciplinary Department of Medicine, University of Bari Aldo Moro Bari Policlinico Hospital, Bari, Italy; 4 IMM CNR, Institute of Microelectronics and Microsystems, National Research Council, Rome, Italy

**Keywords:** Hypoxanthine, Post-mortem interval, Post-mortem biochemical markers, Thanatochemistry, Biofluids, Forensic pathology

## Abstract

The estimation of post-mortem interval (PMI) is of utmost importance for forensic pathologists due to its implication in medico-legal evaluations. Research over the last thirty years has sought new methods for estimating the time of death, particularly focused on human biomarkers whose concentration changes over time after death. Although most studies are based on potassium (K^+^) concentrations in blood and vitreous humor (VH), hypoxanthine (Hx) has shown great promise in assessing PMI. Following PRISMA guidelines, this systematic review addresses the PICO question: "In human cadavers, what is the role of hypoxanthine, where, and with what analytical techniques is it currently used for post-mortem interval estimation?". Twenty-four papers were retrieved. The results indicate that Hx concentration can be estimated in various biofluids, VH being the most commonly accounted for. Furthermore, different pre-analytical procedures are resorted to for sample preparation, such as several methodologies utilized to detect Hx concentration. The relationship between the so-obtained Hx levels and PMI is expressed quantitively (through regressions or correlation coefficients) or semi-quantitatively (by changes in nuclear magnetic resonance spectra). PMI estimation accuracy improves significantly when additional factors are considered (such as ambient and rectal temperature, urea concentration, body weight, and cause of death) or when new methodologies providing flexible regression models are applied. Despite the promising potential, many limitations remain. Notably, the heterogeneity of sample selection and pre-analytical/analytical phases leads to inconsistent results. Thus, much more should be done to lay procedural standards and optimize biochemistry and Hx utilization in PMI-related forensic investigations.

## Introduction

Post-mortem interval (PMI) is defined as the elapsed time between death and the investigation on the body. Its estimation is of paramount importance due to its informative potential within legal-forensic inquiries. However, establishing the PMI continues to be one of the more difficult tasks in the field of forensic sciences and criminal investigation [1–5]. Indeed, despite the vast amount of research conducted, there is still a need for more reliable and precise approaches [6].

The methodologies currently applied in forensic practice are cooling models [7], entomological analyses [8], and biochemical methods [9], the first two of which — despite inherent limitations (especially within entomology) — are the only satisfying Daubert standards, thus meeting the criteria for scientific evidence admissibility in the courts [10, 11]. Meanwhile, new experimental approaches are under development, including studies on the mechanical excitability of human skeletal muscle, iris imaging, and metabolomics [12, 13]. It is well known that cooling methods move from empirical evaluations, such as the body rectal temperature evaluation [5, 14–24], influenced by body weight, environmental temperature, or antemortem hyper- and hypothermia. For this reason, different nomograms have been proposed to estimate PMI by means of body cooling, basically considering weight, rectal temperature, and environmental temperature. However, this method should only be used until the cadaveric temperature is leveled with the ambient one.

Post-mortem biochemistry, or thanatochemistry, is an ascending research field and concerns post-mortem biochemical markers (BPMs) in biological matrices (tissues or fluids) that change over time, correlating with the PMI [25, 26].

Their use in forensic practice requires consideration of inter-individual variability influencing the inherent biological complexity (e.g. differences related to the deceased’s age, gender, cause of death, biological background, lifestyle, medication or drug use, degree of autolysis and putrefaction, environmental temperature/conditions, the survival time, and the PMI) [6, 23, 27–29], but also that these analyses were originally designed and validated for *in-vivo* conditions. This discrepancy significantly complicates the accurate interpretation of biochemical markers in a postmortem context, just like their use as evidence in court. For these reasons, the practical applicability of these approaches in forensic contexts is still limited.

Consistent with the above, additional factors that may reduce the reliability of biochemical methods for PMI estimation include the type of matrix/sample used, the site where the sample is collected, the analytical technique employed, and the statistical methodology applied [4, 17, 30–36].

Moreover, some metabolic changes occurring post-mortem have been identified and attributed to the agonal period of anoxia, the biochemical changes continuation in the early post-mortem period, and easily diffusible substances distribution between erythrocytes and plasma as well as between interstitial fluid, tissue cells, and the blood.

The first studies have been performed on blood [16], cerebrospinal fluid (CSF) samples [17], and above all on the vitreous humor (VH) [18, 24]. VH has been especially investigated due to its inherent anatomical protection determining slower autolytic changes and preservation from external physical (e.g., head injuries) and biochemical conditions (e.g., bacterial contamination and putrefaction), thus extending the time for PMI estimation [18–20, 34, 37–45].

In recent years, several studies have demonstrated the usefulness of VH in PMI estimation by determining changes in its hypoxanthine (Hx) and potassium (K^+^) concentration levels [5, 21, 22, 32, 46], mainly by techniques such as high-performance liquid chromatography (HPLC) [18, 22, 32, 47, 48] and liquid chromatography coupled to tandem mass spectrometry (LC–MS/MS) [44, 49, 50].

Evidence suggests that Hx concentrations increases linearly during the first 24 h after death in both humans and animals [21, 25, 51–53], thus indicating that this analyte could play a pivotal role among BPMs in PMI determination [54–57].

Hx is an intermediate product in the purine catabolism pathway [58]. Before death, when oxygen is available, Hx is oxidized by xanthine oxidase into uric acid, which is the final product of human purine metabolism. In mammals, xanthine oxidase is located in the liver and in the small intestine mucosa. Due to the absence of this enzyme in the blood, the final product of purine catabolism in the bloodstream is Hx, which undergoes liver uptake and is converted to uric acid and excreted.

The Hx normal concentration in living individuals’ blood is 0.5–11 μmol/L^−1^ [58, 59]. In the context of pathologies determining tissue hypoxia, high Hx concentrations are found in blood, cerebrospinal fluid, and urine [48, 58, 60–65] because of the following mechanisms: increase of adenosine monophosphate (AMP), decreased conversion of Hx into uric acid, inhibition of xanthine oxidase enzyme [58].

To the best of our knowledge, no updated literature reviews specifically addressed the role of [Hx] in PMI estimation. Following the PRISMA flow diagram, we have revisited the relevant literature and critically analyzed it to perform a systematic review and inform the ongoing laboratory research on the following main topics: Hx concentration kinetics in the cadaver; methods for PMI estimation employing Hx concentration; Hx measurement techniques; accuracy of Hx-based PMI estimation. At the same time, the most frequently examined biological samples, the statistical analyses performed, the intrinsic and extrinsic variables affecting results, and the effectively explored time after death have been inferred and argued.

## Materials and methods

This review followed the PRISMA (Preferred Reporting Items for Systematic Reviews and Meta-Analyses) protocol [66]. The articles were selected without any time restrictions from PubMed, Scopus, Web of Science, and Cochrane Library databases and by the query: (“hypoxanthine”) AND (“post-mortem interval” or “postmortem interval” or “PMI” or “time since death” or “time from death” or “time of death” or “time after death”). This query is intended to respond to the following PICO (Population, Intervention, Comparison and Outcome) question [67]: “In human cadavers, what is the role of hypoxanthine, where, and with what analytical techniques is it currently sought for the post-mortem interval estimation?”. Articles not published in English, with non-eligible formats (such as case reports, reviews not including research articles, or conference proceedings), and non-focusing on hypoxanthine were excluded. Articles were selected progressively, reading the title, the abstract, and, finally, the full text. Each article eligibility assessment was made independently by all authors and any disagreements were resolved by consensus. Data were extracted from each study and organized into a table, pointing out detailed information about types of study, population, pre-analytical and analytical processes, detection methods, statistical analysis, and the main results.

## Results

### Data extraction

**Fig. 1:**
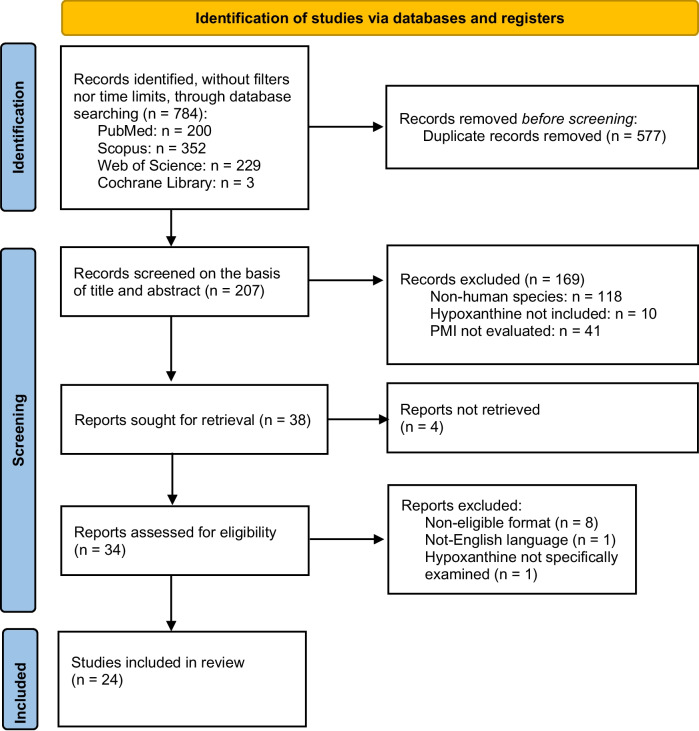
PRISMA Flow Chart

A total of 784 articles were retrieved from database search. After duplicate removal, 207 remaining articles underwent title and abstract screening, leading to the exclusion of an additional 169 entries. The remaining 38 papers were retrieved for full-text review; however, the full text for 4 articlescould not be obtained. Out of the 34 read articles, 24 were included in the review. The last ten articles were excluded due to inadequate article type (8 articles), English language not available (1 article), and lack of specifical analysis for [Hx] in PMI estimation (1 article). The article extraction process is reported in Fig. 1.

Tables 1 and 2 provide the details of the 24 selected articles. Among these, there were 19 research articles, two systematic reviews plus research studies, two technical reports, and one short communication. Of these, 18 were performed in Europe, two in the UK, one in China, one in South Korea, one in Australia, and one in New Zealand.

**Table 1:** Information included in the papers selected for review

**Authors and Year of publication**			**Title**			**Type of article**			**Place the study was performed**				T**issues and Organs**	
[52] Rognum T.O., et al 2016			Estimation of time since death by vitreous humor hypoxanthine, potassium and ambient temperature			Research article			Norway				VH	
[49] Muñoz Barús J.I., et al 2010			PMICALC: An R code-based software for estimating post-mortem interval (PMI) compatible with Windows, Mac and Linux operating systems			Research article			Spain				VH	
[50] Lendoiro E., et al., 2012			Applications of Tandem Mass Spectrometry (LC–MSMS) in estimating the post-mortem interval using the biochemistry of the vitreous humour			Research article			Spain				VH	
[68] Febrero Bande M., et al 2023			IPMICALC: an Integrated Post‑mortem Interval Calculator			Technical report			Spain and Portugal				VH	
[39] Zelentsova E.A., et al 2020			Post‑mortem changes in metabolomic profiles of human serum, aqueous humor and vitreous humor			Original article			Russia				Serum, AH, VH	
[69] Chighine A., et al 2023			Metabolomics investigation of post-mortem human pericardial fluid			Original article			Italy				Pericardial fluid	
[23] Rognum T. O., et al 1991			A new biochemical method for estimation of postmortem time			Research article			Norway				VH	
[20] Madea, et al 1994			Hypoxanthine in vitreous humor and cerebrospinal fluid – a marker of postmortem interval and prolonged (vital) hypoxia? Remarks also on hypoxanthine in SIDS			Research article			Germany				VH and CSF	
[70] Carpenter K.H., et al 1993			Vitreous humour and cerebrospinal fluid hypoxanthine concentration as a marker of pre-mortem hypoxia in SIDS			Research article			United Kingdom				VH and CSF	
[48] Muñoz Barús J.I., et al 2002			Improved estimation of postmortem interval based on differential behaviour of vitreous potassium and hypoxantine in death by hanging			Research article			Spain				VH	
[71] Madea B, Rödrig A 2006			Time of death dependent criteria in vitreous humor – Accuracy of estimating the time since death			Systematic review + Research article			Germany				VH	
[72] Muñoz Barús J.I., et al 2008			Flexible regression models for estimating postmortem interval (PMI) in forensic medicine			Research article			Spain				VH	
[56] Donaldson A.E. and Lamont I.L 2013			Biochemistry Changes That Occur after Death: Potential Markers for Determining Post-Mortem Interval			Research article			New Zealand				Blood	
[73] Pérez-Martìnez C., et al 2017			Quantification of nitrogenous bases, DNA and Collagen type I for the estimation of the postmortem interval in bone remains			Research article			Spain				Bone	
[74] Cordeiro C., et al 2018			A reliable method for estimating the postmortem interval from the biochemistry of the vitreous humor, temperature and body weight			Research article			Portugal				VH	
[55] Go A., et al 2019			Analysis of hypoxanthine and lactic acid levels in vitreous humor for the estimation of post-mortem interval (PMI) using LC–MS/MS			Research Article			South Korea				VH	
[75] Pérez-Martìnez C., et al 2019			Influence of the nature of death in biochemical analysis of the vitreous humour for the estimation of post-mortem interval			Research article			Spain				VH	
[76] Liao L., et al. 2020			An electrochemical biosensor for hypoxanthine detection in vitreous humor: A potential tool for estimating the post-mortem interval in forensic cases			Research article			China				VH	
[54] Bonicelli A. et al 2022			The ‘ForensOMICS’ approach for postmortem interval estimation from human bone by integrating metabolomics, lipidomics, and proteomics			Research article			United Kingdom				Bone	
[47] Passos M.L.C., et al. 2009			Estimation of postmortem interval by hypoxanthine and potassium evaluation in vitreous humor with a sequential injection system			Research Article			Portugal				VH	
[57] Da Cunha E.C., et al 2022			The biochemistry of the vitreous humour in estimating the post-mortem interval – a review of the literature, and use in forensic practice in Galicia (northwestern Spain)			Systematic review + Retrospective study (research paper)			Spain				VH	
[32] Muñoz JI., et al 2006			A high-performance liquid chromatography method for hypoxanthine determination in vitreous humour: application to estimation of post-mortem interval			Short communication			Spain				VH	
[21] Camba A., et al 2014			High variation in hypoxanthine determination after analytical treatment of vitreous humor samples			Technical report			Portugal				VH	
[22] James, R.A., et al 1997			Determination of postmortem interval by sampling vitreous humour			Original article			Australia				VH	
**Number of samples (research articles) / Number of papers (reviews)**	**Exclusion (research articles) / selection (reviews) criteria**			**Information about population** **(sex, age, cause of death, etc.)**			**Ambient temperature at the time of tissue/organ sampling**	**PMI assessed**			**Pre-analytical processes**			**Detection methods**
n: 132 + 17 live controls obtained from vitrectomies [52]	None			Sex Males (n: 66) Females (n: 66) Cause of death - 8 accidents - 2 homicides - 4 suicides - 118 natural			n: 34 at 5 °C n: 18 at 10 °C n: 18 at 15 °C n: 55 at 23 °C	17 min – 118 h			Samples were centrifuged at 9000 rpm for 90 min at 4 °C Then, they were stored at −20/−75 °C until measurement			Capillary electrophoresis for Hx Flame photometry for K^+^
n: 201 [49]	None			Distinctions based on sex, age, and cause of death (and medical care) were performed, but numbers of each category are not provided			Not available	Not available			/			Not available
n: 20 (for validation) n: 15 (for effect of contamination) [50]	None			Not available			Not available	8,6 h – 16,5 h			Centrifugation at 14.500 rpm To evaluate the effect of blood contamination, specimens were contaminated with rising blood concentrations (1, 2, 3, 4 and 5 µL)			HPLC MRM
n: 532 331 from Coimbra University; 201 from Santiago de Compostela University [68]	/			Following information about samples were registered, although not specified in the paper: sex, age, bodyweight, PMI, rectal temperature, ambient temperature at moment of sampling, cause of death, and concentration of K^+^, Hx, and urea			Not available but known	Not available but known			/			IPMICALC
n: 31 blood samples n: 33 AH samples n: 33 VH samples [39]	From the initial 38 post-mortem donors, following exclusion criteria were applied: - death from cancer - blood sample coagulated or contaminated			Not available			15 °C before sampling (for 3 – 30 h)	5 h – 58,6 h			Cadavers taken at 15 °C for 3 h – 30 h before sampling Then, centrifugation of samples was performed: - centrifugation of blood samples: 3000 rpm for 10 min - centrifugation of AH samples (13200xgr, 30 min, + 4 °C) - centrifugation of VH samples (+ 4 °C for 30 min)			^1^H-Nuclear Magnetic Resonance spectrometer
n: 24 Subdivision in TEST set (n:6) and TRAINING set (n:18) [69]	Exclusion criteria: - pathological pericardial effusion - macroscopic blood contamination			Sex M/F ratio: 1:3 Age 20–87 y.o Causes of death Identified but not specified			Not available before sampling	16 h – 170 h			Samples were stored at −80 °C after collection. Centrifugation for 10 min at + 4 °C was performed An aliquot of 500 µ underwent ultrafiltration Another aliquot of 500 µ underwent liquid–liquid extraction			^1^H- Nuclear Magnetic Resonance spectrometer was performed
n: 87 [23]	None			Sex Males (n: 54), Females (n: 33) Age 15–98 y.o. (mean: 71,5 y.o.) Cause of death - myocardial infarction (n:53); - accidents (n: 139); - various other conditions (n:21)			5 °C (n:33), 10 °C (n:16), 15 °C (n:16) or 23 °C (n:23)	Up to 120 h			/			HPLC for Hx Flame photometry for K^+^
For VH n: 92 + n: 43 (withdrawn at timed intervals between 2 and 20 h) For CSF Not specified [20]				Causes of death were known: natural causes with a brief terminal episode or traumatic causes with a brief terminal episode			Not available	Known but not specified			Samples of VH were taken from both eyes, in some samples at timed intervals between 2 and 20 h Samples of CSF were taken through a single puncture			HPLC for Hx Ion selective electrodes for K^+^
For VH n: 119 For CSF n: 75 [70]				For VH Age Children aged from 1 week to 2 y.o Cause of death - unexplained = SIDS (n: 68) - severe cardiac/pulmonary disease (n: 13) - other known causes (n: 38) For CSF Cause of death - SIDS (n: 45) - Others (n: 30)			Not available	2,5 h – 120 h			Samples were stored at −20 °C before analysis After that, centrifugation was performed			HPLC was modified after Morris et al
n: 206 Samples were taken from 176 corpses. In 30 cases, samples were taken from both eyes [48]	Exclusion of the following samples: - time of death couldn’t be established to within ± 15 min - non-transparent specimens - new-born infants (< 6 months)			Sex Males (n: 141), Females (n: 35) Subgroups of cause of death Hanging deaths (n: 35); non-hanging deaths (n: 141) Other factors Age and medical care were known, although not reported			Not available	1,08 h – 28,91 h			Samples were stored at 4 °C before analysis, for a maximum of 48 h Furthermore, centrifugation was performed at 3000 rpm for 10 min			
n: 198 (64 own cases + 134 cases taken from Munoz et al.) [71]	Exclusion of not crystal-clear VH samples			Cause of death, simple acquisition, pre-treatment, and analytical procedures were known to be identical between own cases and Munoz et al. cases			Not available	0 h – 96,8 h			Samples were frozen at −70 °C before determination Centrifugation for 10 min at 3000 rpm was applied			HPLC–MS
n: 201 [72]	The following samples were excluded: - non-transparent samples - samples from infants aged less than 6 months			Information about sex, age, medical care, and cause of death has been registered, although data are not reported			Not available	1 h – 29 h			Centrifugation at 3000 rpm for 10 min Storage at 4 °C before analysis for a maximum of 24 h was provided			K^+^ was analyzed via BM/747 analyzer (Boehringer Mannheim) Hx was analyzed via Waters 996 PDA system with revers-phase column
n: unknown for human blood samples [56]	/			/			Not available	0 h – 96 h			Human blood samples were incubated alongside animal corpses			Enzymatic fluorescence assay kit for Hx
n: 80 (among them, 73 femur, 5 tibia, 2 humerus) Samples were collected from crypts organized in columns to avoid contact with the ground, thereby minimizing transformation and diagenesis processes [73]				Sex Males (n: 50), Females (n: 50) Age Age: 20 – 97 y.o			Not available	5 years – 47 years			Bones were demineralized, then samples were centrifuged for 10 min at 4000 rpm			For quantification of Hx and other nitrogenous bases, HPCL was performed For DNA extraction and quantification, the casework protocol was used For proteins, HPCL/MS/MS was used
n: 331 [74]	Blood contamination of VH was considered through MRM transition. Consequently, contaminated samples were excluded			Cause of death Sudden or unexpected death (not better specified) Weight 35 – 127 kg Post-mortem rectal temperature 22,2 – 37,8 °C Ambient temperature at the time of sampling 9,3 – 29,4 °C Whether or not resuscitation maneuvers were performed Specified			Considered but not available	1,50 h – 22,50 h			Samples were centrifugated for 10 min at 14,500 rpm			For hypoxanthine, xanthine, and guanine detection, LC-MSMS was performed K^+^ and Na^+^ were quantified through the indirect potentiometry method
n: 79 [55]	/			Sex Males (n: 53), Females (n: 26) Age Mean age: 51 y.o			The samples were subdivided in temperature range (−10 – 5 °C; 6 – 15 °C: 16 – 25 °C: 26 – 32 °C) Average daily ambient temperature from −9,5 °C to + 31,8 °C	13 h – 103 h			For Hx sample preparation, the sample was centrifuged at 1177 g for 10 min			HPLC and LC–MS/MS for both Hx and L-lactic acid
n: 250 [75]	/			Sex Males (n: 174), Females (n: 76) Age 58,75 ± 20,84 Cause of death - natural (n: 148; among them: 70 heart affection, 20 hanging, 3 drowning, 7 suffocation, 6 suffocation, 42 others) - violent (n: 102; among them: 66 multiple injuries, 30 violent asphyxias, 6 intoxications) Prior medical pathologies Known, but not specified			Not available	2 h – 24 h			Samples were stored at −72 °C, then centrifuged for 10 min at 3000 rpm at 4 °C before analysis			HPLC–UV for Hx Multichannel Autoanalyzer for K^+^ and uric acid
n: 4 [76]	Subjects with eye trauma were excluded			Cause of death Vehicular accident cases			12 °C, 16 °C, 18,5 °C, 26 °C	9 h – 46 h			Samples were kept at −80 °C before sampling			CNF and XOD/GA/PDA-CNF’s modified GCE were prepared. The structure of this electrochemical biosensor was analyzed through FTIR and electronic microscopy, then its efficiency was studied in solution with different [Hx] Finally, samples of VH from 4 cadavers (previously stored at −80 °C) were used to quantify VH through XOD/GA/PDA-CNF electrochemical biosensor
n: 24 [54]				4 female donors. Samples were taken in a pre-deposition phase (12 samples, 4 for each donor at 3 different PMIs) and a post-decomposition phase (12 samples, 4 for each donor at 3 different PMIs)			Not specified	2 days – 872 days (2 – 3 – 10 – 10 – 219 – 790 – 834 – 872 days)			Samples were kept at 4 °C before sampling, and then placed outdoors to decompose Taken samples were transferred at −80 °C freezer. After that, they were shipped on dry ice and then transferred to another freezer at −20 °C. After defrosting, a powder was obtained through Dremel drill (5000 rpm). The powder was stored at −80 °C before testing Centrifugation was performed twice during the extraction			LC–MS
Unknown [47]	/			/			Not specified	Not specified						SIA (Sequential Injection Analysis): for K^+^, it performs potential measurements with a decimillivoltimeter micropH 2022, a home-made tubular electrode, and a reference electrode; for Hx, spectrophotometric measurement with Thermo-Spectronic Helios gamma-UV spectrophotometer is performed The results of SIA in Hx (and K^+^) detection are compared with classic methods (spectrophotometry and ion-selective electrode, respectively for Hx and K^+^)
In the reviewn: 36 selected papers 227 papers were identified by searching and matching different keywords on PubMed (15 keywords) and Google Scholar (4 keywords) In the retrospective cohort study n: 1768 [57]	In the review The following exclusion criteria for the selection of papers were applied: - non-human samples - use of biomarkers other than K^+^ and Hx - no correlation with 95% CI - hospitalization before death - PMI over 210 - not aiming to measure the accuracy of PMI estimates For the retrospective cohort study A prolonged period in the hospital and a question about the reliability of the information was considered an exclusion criterion			In the retrospective cohort study Sex 1256 males, 512 females Causes of death - cardiovascular (35,69%) - asphyxiation (15,78%) - traumatic accidents (14,71%) - deaths from falls (12,10%)poisoning (6,11%), wound from weapons (3,28%) - respiratory system (2,94%) - digestive system (2,60%) - indeterminate (2,38%); - neurological causes (2,04%) - malignancies (0,79%) - thermal deaths (0,45%) - metabolic (0,23%)CKD (0,28%) The following groups of cause of death were discriminated: natural (n: 973), accidental (n: 495), suicide (n: 285), and homicide (15)			/	Not specified (retrospective cohort study)			/			Not specified
n: 134 [32]	None			Not specified			Not specified	Not specified			Samples were stored at −20 °C until analysis			HPLC (Waters HPLC system)
n: 105 [21]	None			Not specified			Not specified	Not specified			Firstly, samples were centrifuged at 1,650 for 8 min at 15 °C. After that, different pre-analytical processing was tested: enzymatic digestion (crystals of hyaluronidase), heat, sonication, and centrifugation Then, samples were frozen at −20 °C until analysis and centrifuged at 13,100 rpm for 10 min			Solid Phase Extraction (SPE) and LC–MS/MS
n: 100 [22]	The exclusion criteria were the following: - unknown PMI - subject required for cornea donation 55 samples were not completely clear. Nevertheless, they were not excluded			Sex Not recorded Age 8–89 y.o. (mean age: 50,1 y.o.)			Not specified	Not specified			Storage at 4 °C before analysis, but some of the samples were stored at higher temperatures (no homogeneity)			HPLC and ultraviolet light (250 nm) for detection
**Statistical analysis and validations (research articles) / Methods applied (reviews)**		**Further parameters considered for statistical analysis**			**Results**							**Other analytes-related findings**		
Statistical analysis using IBM SPSS Statistics Version Linear multivariable regression was provided [52]		Ambient temperature			PMI: Hx × 0,215 + T x (−0,467) + Hx x T x (−0,005) + 10,353 Median [Hx] live patients’ VH: 0,444 (range: 0,302–0.759) µmol/L Median concentration of [Hx] in VH: 115.5 mmol/L (6–554 mmol/L) The standard error of curves is not linear, with the lowest standard error for [Hx] around 150 µmol/L, corresponding to a 95% confidence interval of 2,5 h A good corresponding between post-mortem interval values estimated from VH [Hx] and [K^+^] was found, with a correlation (r^2^) of 0,89							For K + PMI = K + × 5.164 + T × 0.174 + K + x T x (−0.100) −19.588 Median [K^+^] in live patients’ V: 4,5 (range: 4,2–5,1) mmol/L Median concentration of [K^+^] in VH: 10.8 mmol/L (4.4–28.2 mmol/L) The standard error of curves is not linear, with the lowest standard error for K^+^ around 12 mmol/L, corresponding to a 95% confidence interval of 3 h		
PMICALC (R-code-based software with Additive Models (AM) and Support Vector Machines (SVM) Validation of the model was performed estimating the mean-square error [49]		/			The results, in terms of Mean Square Errors from 10,000 replications, showed an average of 0.20 for classical linear regression, and 0.16 for both AM and SVM. Additionally, the medians were 0.20 for linear regression, 0.16 for AM, and 0.15 for both AM2 and SVM							Combination of Muñoz-Barús et al. methods for determining PMI from [U], [K^*^], and [Hx] in VH		
Data were acquired using MRM (Multiple Reaction Monitoring) mode Linearity was assessed by analyzing four calibration curves prepared on different days, utilizing least-squares regression Additionally, evaluations were conducted for LOD (Limit of Detection), LOQ (Limit of Quantification), imprecision, analytical recovery, extraction efficiency, process efficiency, and matrix effect [50]		/			Using least-squares regression, linearity with a coefficient r^2^ of 0,99 was obtained The following values for Hx came out: LOD 2,5 µM; calibration range: 10–200 µM; intercept ± SD: 20.74 18.10; slope ± SD: 5.16 ± 3.67; r^2^ ± SD: 0.991 ± 0.0025							For guanine LOD: 0.25 µM; calibration range: 1–40 µM; intercept ± SD: 1.74 ± 3.05; Slope 1 ± SD: 6,23 ± 3,30; r^2^ ± SD: 0,0026 ± 0,0031 For xanthine LOD: 0,5 µM; calibration range: 2–40 µM; Intercept ± SD: 2,41 ± 2,32; Slope ± SD: 0,81 ± 0,36; r^2^ ± SD: 0,9934 ± 0,0025		
The IPMICALC provides an estimation of PMI by adopting the formulas developed by Cordeiro et al., such as models of Munoz et al. (AM and SVM models) Estimates of PMI provided have 95% confidence intervals [68]		Cordeiro et al. models consider rectal temperature, ambient temperature, [Hx], [K^+^], [U], and body weight Munoz et al.’s AM and SVM models consider [K^+^], [Hx], and [U]			/							[U] and [K^+^] are considered, if available		
A correlation analysis between PMI and the concentration of 42 metabolites was performed p values corrected by using False Discovery Rate (Benjamini–Hochberg procedure) were calculated [39]		/			Linear correlations of [Hx] in serum (r: 0,55), AH (r: 0,63), and VH (r: 0,62) with PMI were found Models with RMAE 0,6 for Hx							Multivariate linear regression not including [Hx] (PMI(AH) = 0.0091 × [Creatine] + 0.081 × [Choline] + 0.18 × [Betaine] + 3.2), because it considered the three analyzed metabolites with higher R-value R values of other metabolites are described		
Exploratory data analysis was conducted using PCA (Principal Component Analysis). Regression models for estimating PMI from quantified metabolites were developed using oCPLS2 (orthogonally Constrained PLS2). Relevant predictors were identified through stability selection using VIP (Variable Influence on Projection) [69]		/			Predictive score-regression model for ultrafiltrated samples - R^2^: 0,547 - Q^2^: 0,244 - SDEC: 30 h - SDECV: 38 h - SDEP: 33 h Much linearity emerged when a PMI greater than 100 h is excluded: - R^2^: 0,543 - Q^2^: 0,444 - SDEC (standard dev): 17 h - SDECV: 19 h - SDEP: 16 h Predictive score-regression model for liquid–liquid extracted samples - R^2^: 0,669 - Q^2^: 0,161 - SDEC (standard dev): 25 h - SDECV: 41 h - SDEP: 34 h Much linearity emerged when a PMI greater than 100 h is excluded: - R^2^: 0,701 - Q^2^: 0,569 - SDEC (standard dev): 14 h - SDECV: 17 h - SDEP: 13 h							A regression model includes other metabolites, but no formula is available		
Construction of scatterplots for every temperature group. The median slope was drawn [23]		Ambient temperature			[Hx] and [K^+^] increased linearly with time. Distinguishing four temperature groups, different median [Hx] increases emerged: 4.2 µmol/l/h, 5.1 µmol/l/h, 6.2 µmol/l/h and 8.8 µmol/l/h for 5, 10, 15 and 23 °C, respectively Significant correlations between vitreous [Hx] and [K^+^] levels were found. (r: 0,93)							Potassium median increase was 0.17 mmol/l, 0.20 mmol/l, 0.25 mmol/l, and 0,30 mmol/l for 5, 10, 15 and 23 °C groups, respectively The slopes for each temperature group were the following: 0,17; 0,2; 0,25; 0,3 for 5 °C, 10 °C, 15 °C and 23 °C, respectively		
Construction of scatterplots with 95% CI [20]		/			[Hx] in VH and correlation with PMI: r: 0,7138; a: 3,6991; b: 1,293; 95% confidence limits: ± 30,1 h These data were not specified for CSF, but exponential rise of Hx is evident, with the steepest slope appearing in the first 15 h and an equilibration reached after 15–20 h							K^+^ in VH and correlation with PMI: r: 0,925; a: 6,3177; b: 0,179; 95% confidence limits: ± 16,3 h These data were not specified for CSF, but an exponential rise of K^+^ is evident		
Correlation analysis using Spearman rank sum test Regression analysis calculated by least squares analysis Comparison between group by using Mann-Witney U test [70]		/			[Hx] in VH shows an increase at a mean rate of 8,3 µmol/l/h in the first 24 h post-mortem, with a correlation coefficient (r) of 0,46. Beyond 24 h after death, the correlation of [Hx] in VH with PMI becomes weaker (r: 0,32) [Hx] in CSF showed no significant correlation with PMI in any timespan Median [Hx] in VH (normalized to a 24 h equivalent PMI) resulted 338 µmol/l in case of SIDS; 264 µmol/l in case of cardiac/pulmonary disease; 295 µmol/l in case of other causes of death. Median [Hx] in CSF was 884 µmol/l in case of SIDS; 796 µmol/l in case of cardiac/pulmonary disease; 796 µmol/l in case of other causes of death. Although, no significant difference between groups emerged							None		
Simple linear regression was elaborated using SPSS 9.0.1 for WindowsTM Testing the goodness-of-fit was carried out by calculating Cook’s distance and using the Durbin–Watson method [48]		Urea concentration Hanging or non hanging as a cause of death			Linear regression formulas were obtained [HX] = 26,459 + 3,017PMI (R^2^ = 0,518) Then, an evaluation after stratification for hanging or non hanging deaths was provided, obtaining the following regressions For hanging group: [Hx] = 15,161 + 4,957PMI, R^2^: 0.757 For non-hanging group: [Hx] = 28,155 + 2,733PMI, R^2^: 0.486 Then, the variation of regression was evaluated considering values of urea ≥ 30 mg/dl, so obtaining other formulas with lower R^2^ values For hanging group: [Hx] = 17,438 + 4,567PMI, R^2^: 0.764 For non-hanging group: [Hx] = 30,123 + 2,573PMI, R^2^: 0.457 By changing variables, R^2^ values (1-R^2^) were higher, indicating a better precision							The same evaluation and formulas were carried out for [K^+^] in VH, firstly evaluating the full cohort, then after stratification based on hanging or non hanging as a cause of death. Finally, another stratification was made based on urea concentration		
Pitman’s T test (α = 5%) was used to check statistical significance The accuracy of predictions using [Hx] as dependent or independent variable has been assessed [71]		Ambient temperature			By changing the formula using vitreous [Hx] as independent variable to predict PMI, accuracy (95% confidence limit) changed, as the error rose from 15,89 h to 13,69 h. Different values were obtained in rise PMI/Hx (0,22 with PMI as dependent variable, 0,29 as independent variable), intercept (−2,24 and −7,85, respectively), and standard deviation (6,98 and 8,11, respectively). R^2^ is 0,742 in each case Comparing separately the “own samples” group and the “Munoz et al.” group, the rise in accuracy was greater in the latter: the error sank from 12,57 h to 9,09 h							16 formulas including [K^+^] are reported^.^ The influence of gender, age of deceased, cause of death, ambient temperature, and other factors in PMI estimation through [K^+^] in samples is assessed Other statistical analyses have been performed to evaluate the validity of Loess procedure for the estimation PMI though [K^+^]		
Application of GAM (Generalized Additive Models) and SVM (Support Vector Machines) for the estimation of PMI. Results were compared with those obtained using LR (Linear Regression) methods For quality control, a m-fold cross-validation model was applied to assess the error for regression [72]		Cause of death (hanging or non-hanging), [U], [K^+^]			For linear regression The following formula is elaborated: log(PMI) = β_o_ + β_1_[K^+^] + β_2_[Hx] + β_3_[U] + ε For Additive Models: log(PMI) = α_o_ + f_1_[(K^+^)] + f_2_[(Hx)] + f_3_[(U)] + ε Following values are obtained regarding this model: R^2^ of 0.812; 13.9 estimated degrees of freedom; 83.9% of deviance explained by this model Incorporating the cause of death into the additive model (AM2), with cause of death (D) as a binary variable (hanging, non hanging) $Log\left(PMI\right)=\beta_1+\beta_21_{D=h}+f_3\left(\left[U\right]\right)+f_{1h}\left(\left[K^+\right]\right)1_{D=h}+f_{2h}\left(\left[Hx\right]\right)+f_{1\overline h}\left(\left[K^+\right]\right)1_{D=\overline h}+f_{2\overline h}\left(\left[Hx\right]\right)1_{D=\overline h}+\varepsilon$ This model has a R^2^ of 0.831, an estimate degree of freedom of 19.2, a deviance of 86.4% For Support Vector Machine $\log\left(PMI\right)=f_w\left(\left(\left[K^+\right],\;\left[Hx\right],\;\left[U\right],\;D\right)^t\right)+\varepsilon=\sum_{i=1}^n\beta_ik\left(\left(\left[K^+\right],\;\left[Hx\right],\;\left[U\right],\;D\right)^t,\;{\left(\left[K^+\right],\;\left[Hx\right],\;\left[U\right],\;D\right)}_i\right)+\varepsilon$ with k being a radial basis Gaussian kernel The calculated mean-squared error was 0.045							The model includes [K^+^] and [Urea]		
Average concentrations and standard deviations were calculated using ANOVA, followed by a post-hoc Turkey analysis performed using R studio [56]		/			Not available							pH and other analytes (lactic acid, formate, NAD^+^ and NADH, nitrogenous compounds, uric acid) were considered		
The methods were validated for linearity, LOD, LOQ, imprecision, analytical recovery, extraction efficiency, process efficiency, matrix effect SPSS 20.0 for statistical analysis. Spearman’s coefficient was used to study numerical and binary qualitative variables. Then, Kruskal–Wallis test was applied to analyze differences between groups. Different models of regression were examined to represent PMI as dependent variable. Finally, discriminant analysis was used to assess the capacity to classify a sample at different PMI. In each analysis, two different groups were distinguished (PMI < 20 years and ≥ 20 years) [73]		Age			Values obtained for [Hx] show a mean concentration of 3,732 ± 2,594 in the first group (PMI < 20 years) and of 3,320 ± 2,421 in the second group (PMI ≥ 20 years). The calculated correlation factor is negative for both groups (−0,375 for the first, −0,457 for the second, with a p > 0,05 just for the latter). In the discriminant analysis, a correct classification was obtained in 95% cases of the first group, in 80% cases of the second group For Hx, the predicted group membership is 45% for PMIs < 20 years, 68% for PMIs ≥ 20 years, with a percentage of correct classification of 57,8% The curvilinear regression obtained for [Hx] in bones in case of PMI < 20 years follows an exponential model, with following values: R of 0.501, R^2^ of 0.251, p of 0.024. The obtained equation is the following: y = 9,007 × exp (−0,104 × x)							The same analysis was carried out for other analytes: DNA, adenine, guanine, purines, cytosine, thymine, pyrimidines, xanthine, and collagen type I		
The analytical method assessed in terms of LOD, LOQ, linearity, precision accuracy and recovery GAMs (Generalized Additive Models) were applied to design models considering multiple variables. For each model, a cross-validation strategy was performed to assess the predictive capacity: samples were divided into two groups, the first (80%) used to build models, the second (20%) to obtain predictions and to compute the mean square error. Finally mean square errors obtained with GAM were compared with those of Henssge’s Nomogram [74]		Ambient temperature, rectal temperature, weight			Model 1: log (PMI) = α + f_1_ (T rectal) + f_2_ (Hx) + f_3_ (K^+^) + f_4_ (U) + f_5_ (weight) + ε Standard error: 0,2391. For all variables, p value < 0,05. R^2^: 0,784. Mean square error: 0,074 Model 2: log (PMI) = α + f_1_ (T rectal) + f_2_ (Hx) + f_3_ (K^+^) + ε Standard error: 0,2801. For all variables, p value < 0,05. R^2^: 0,702. Mean square error: 0,088 Model 3: log (PMI) = α + f_1_ (T rectal) + f_2_ (Tamb) + f_3_ (Hx) + f_4_ (K^+^) + f_5_ (U) + f_6_ (weight) + ε Standard error: 0,2381. R^2^: 0,786. Mean square error: 0,077 Model 4: log (PMI) = α + f_1_ (T rectal) + f_2_ (Tamb) + f_3_ (Hx) + f_4_ (K^+^) + f_5_ (U) + ε Standard error: 2,607. For each variable except T amb, p value < 0,05. R^2^: 0,743. Mean square error: 0,080 Model 5: log (PMI) = α + f_1_ (T rectal) + f_2_ (Tamb) + f_3_ (weight) + ε (similar to Henssge) Standard error: 0,3406. R^2^: 0,529. Mean square error: 0,128 Henssge’s Nomogram (just cross-validation). MSE: 0,215							The models included weight, Tamb, Trec, K^+^, and urea		
The analytical method was validated assessing LOD, LOQ, linearity, recovery, precision, accuracy. Precision and accuracy were obtained through inter- and intra-day analyses Correlation of [Hx] and [Lac] in VH with PMI was corrected by temperature, using a multi-linear regression method. Evaluations were carried out with Pearson’s correlation test [55]		Ambient temperature, seasonality			Validation test for [Hx] resulted in following values: R^2^ of 0.9977; LOD of 10 µM, LOQ of 50 µM, intra-day precision of 5.94–9.96%, inter-day precision of 1.92–4.34%, recovery of 2.46–5.77%, acceptable accuracy between 80 and 120% Correlation between [Hx] in VH and PMI was 0.53 before temperature correction, and 0.59 after temperature correction. After division by temperature groups (−10 – 5 °C; 6 – 15 °C; 16—25 °C: 26 – 32 °C), following values of correlation were found: 0.76, 0.65, 0.51, 0.38. After division by season, the correlation was 0.43, 0.58, 0.49, 0.80 (for spring, summer, autumn, winter, respectively). After correction by season group and temperature, following values were obtained: 0.84, 0.69, 0.88, 0.81 (for spring, summer autumn and winter, respectively)							The same validation test and the same correlations were assessed for [L-lactic acid] Correlation between [L-lactic acid] concentration in VH and PMI was 0.38 before correction by temperature, 0.42 after correction by temperature. After division by temperature groups, following values of correlation were found: 0.71, 0.48, 0.24, 0.28. After division by season, the values were 0.22, 0.28, 0.40, 0.71 (spring, summer, autumn, winter, respectively). After correction by season group and temperature, correlation values were the following: 0.42, 0.29, 0.48, 0.72 (for spring, summer autumn and winter respectively)		
SPSS version 20.0 for arithmetic means and standard deviation. F-test and ANOVA with level of significance of p < 0,05 were applied. Pearson correlation coefficient was used to assess the degree of correlation of analytes’ concentration in VH and PMI. Linear regression analysis was used to obtain equations. At the end, correlation analysis and analysis of variance through multiple linear regression were made [75]		Potassium, uric acid, type of death (violent or natural)			Correlation between [Hx] in VH and PMI is (r) 0.234 (with p < 0,01). Correlation between [Hx] and [K^+^] in VH is (r) 0.293. Correlation between [Hx] and [uric acid] is (r) 0.541- From the linear regression analysis, the following equation was obtained: PMI = 4,946 + 0,397 × K^+^—0,110 × uric acid + 0,166 × Hx (with R^2^: 0,217 and p ≤ 0,0001 In multiple linear regression analysis, for the group of 0–12 h of PMI, R^2^ is 0.361 for natural deaths and 0.462 for violent deaths. For the group of 13–24 h of PMI, R^2^ is 0.157 for natural deaths and 0.268 for violent deaths							Correlation between [K^+^] and PMI is (r) 0.417 (with p < 0,01)		
To assess the efficacy of electrochemical biosensor (XOD/GA/PDA-CNF) in vitreous [Hx] detection, a calibration curve is elaborated Application of formulas of Rognum T.O. et al. formula [PMI = Hx × 0,215 + T x (−0,467) + Hx x T x (−0.005)] for the estimation of PMI, evaluating [Hx] in VH, firstly through a classical automatic biochemistry analyzer, and then though XOD/GA/PDA-CNF electrochemical biosensor The evaluation of LOD was obtained applying the equation C_m_ = 3S_d_/S [76]		Ambient temperature (formula of Rognum et al.)			To assess the efficiency, the following regression was obtained: y = 0,09537 C_Hx_ + 1,217 (R^2^ = 0,997). LOD was evaluated to be 2 µM Recoveries between 91 and 106% were found							No		
For metabolomics Data were normalized by means values. Cube root transformation and Pareto scaling were applied. PCA (Principal Component Analysis) was applied to profiles obtained by each LC–MS separation. Then, univariate analysis was performed by Kruskal–Wallis. Dunn’s test with Holm’s corrected p-valued was also performed to overview differences between different PMIs. Then, PLS-DA was used to analyze each omics block. To check correlation between blocks, PLS regression and DIABLO analysis were applied [54]		/			From the omics integration using PLS regression, a r value of 0.94 between metabolomics and lipidomics, 0.96 between metabolomics and proteomics were found [Hx], such as [creatinine] and [D-Neopterine], were positively correlated with all lipids and with some proteins							Following metabolites were observed in metabolomics profile: creatine, taurine, hypoxanthine, 3-hydroxybutirate, creatinine, phenylalanine) The same procedure was applied to discover proteomic (histones, hemoglobin, ACTB, VIME) and lipidomic (3-lysophosphatidylcholine, phosphatidylcholine, phosphatidylinositol) profiles From the omics integration using PLS regression, a r value of 0.87 between proteomics and lipidomics		
Calibration curves with 95% confidence limits for the intercept and slope were evaluated, along with the linear working range, detection limits, sampling rate, and repeatability for the determination of [Hx] and [K^+^] For evaluation of agreement between SIA and HPLC methods, t-test was carried out [47]		/			Comparing SIA system with classical methods, the following linear relationship was found out for [Hx]: C_SIA_ = (0,951 ± 0,078) x C_HLPC_ + (2,9 ± 7,9) Furthermore, following values were obtained for validation of SIA system for [Hx] detection: R^2^ of 0.998, RSD of 4.03% for 22.95 µmol/l, 4.84% for 31.08 µmol/l From the t-test, a value of −1.02 was obtained for [Hx] Applying SIA method for vitreous [Hx], a r of 0.82522 was obtained At the end, following linear regressions were proposed: C_Hx_ = 7,12 PMI + 31,49 or PMI = 0,14C_Hx_ – 4,42. R^2^ = 0,238							Furthermore, following values were obtained for validation of SIA system for [K^+^] detection: R^2^ of 0,999, RSD of 2,58% for 0,519 mmol/l, 1,05% for 13,1 mmol/l In comparison of SIA system with classical methods, a linear relationship was found out for [K^+^] detection: C_SIA_ = (1,001 ± 0,092) x C_FP_—(0,35 ± 0,84) For the evaluation of agreement between both methods, t-test was carried out, obtaining a value of −3.88 for [K^+^] Applying SIA method for vitreous [K^+^], a r of 0.67814 was obtained. At the end, following linear regressions were proposed: C_K+_ = 0,19 PMI + 6,23 or PMI = 5,36C_K+_ – 33,41. R^2^ = 0,222		
For the review Criteria of SIGN (Scottish Intercollegate Guidelines Network) were applied to each selected article for assessment of to assess level of evidence and degree of recommendation Following data of selected papers were recorded: sample size, extraction, and treatment of VH samples, variable recorded, other analytes, bodyweight, age, rectal temperature, R coefficient, SD, MSE, prediction or correlation formulas, 95% CI for PMI, p value [57]		/			For the review As results, the retrospective cohort studies of Chandrakanth et al., Srettabunjong et al., and Garland et al. were classified as 2- D, while those of Adjutantis et al., Aggarwal et al., Gamero et al., Mulla et al., and Palacio et al. had classifications of 2- C. The remaining 25 studies were classified as 2 + C							K^+^		
Statistical analysis was carried out using Statistix and SPSS (Windows version) using a simple linear regression according to Munoz et al [32]		/			Detection limit: 0,02 µM. Measurement limit: 0.08 µM. The coefficient of variation was 0.04–0.96 and the calibration curve was the following: y = 169 x – 42 (R^2^ = 0,999) The proposed formula is PMI = 0,183 [Hx] + 0,599, with R^2^ = 0,531							No		
Statistical analysis was performed through R software version 2.11.1, applying a natural logarithm transformation. To assess the effect of pre-analytical treatment, a linear mixed-effects model was used. Differences between the four pre-analytical treatments was considered significant at p < 0,005 Non-parametric Wilcoxon test was applied Bland-Atlman Plots were performed to outline hypoxanthine concentration deviations of one pre-analytical method compared with others [21]		Different type of pre-analytical treatment of samples: - enzymatic digestion - heat - sonication - centrifugation			The following statistical parameters were found: minimum and maximum concentration, range, median, median deviation and coefficient of variation for median for each pre-analytical treatment performed As result of non-parametric Wilcoxon test, statistically significant differences were found between enzymatic digestion and heat, between enzymatic digestion and centrifugation, and between enzymatic digestion and sonication. No statistically significant differences were found between sonication and centrifugation, between sonication and heat, and between centrifugation and heat 75% of data obtained after enzymatic digestion are lower than medians of other pre-analytical treatments							No		
A first experimental group was made to elaborate simple linear regressions based on the relationship between PMI and [Hx] or [K^+^] in VH. From these estimations, obtained formulas were applied to the control group to compare PMI estimations to actual values. Analyses of error and variation were performed for each equation [22]					Post-mortem K^+^ and Hx concentrations were correlated (r: −0.54) Based on [Hx], the following regression was calculated: PMI = 0,31 × Hx + 0,05. The intercept of the line on the y-axis was −0.15 µmol/l; slope was 3.2 µmol/l/h Applying the equation to the control group, a mean overestimation of 3.6 h with SD of 19 h was calculated. Using both equation (obtained considering [Hx] and [K^+^]) calculating the mean value of respective results, an overestimation of 2,0 h with a SD of 15 h is obtained Madea et al. equation for PMI estimation from [Hx] in VH implies an average underestimation of 2 h and a SD of 22 h. Rognum et al.’s equation implies an underestimation of PMI of 5 h on average and a SD of 16 h							Based on [K^+^], the following equation was calculated: PMI = 4,32 × K^+^—18,35. The intercept of the line on the y-axis was 4,2 mmol/l; slope was 0,23 mmol/l/h Applying the equation to the control group, a mean overestimation of 0,4 h with SD of 18 h was calculated Sturner et al.’s equation for PMI estimation from [K^+^] in VH has an average underestimation of 9 h and a SD of 30 h		
**Major findings**										**Declared study limitations**				
The rate of increase of [Hx] and [K^+^] was not age-dependent or sex-dependent The slope of the linear correlation is temperature-dependent, as it becomes steeper with increasing temperature In subjects that underwent a significant period of ante mortem hypoxia, [Hx] may be significantly elevated before death [52]										Most cases have PMI shorter than 36 h A large proportion of cases is older than 60 years, which limits the detection of age effect on [Hx] and [K^+^] with increasing PMI Female subjects were older and their PMI was shorter than those of male subjects				
Only [K^+^] and [Hx] levels in VH have a significant relationship with PMI The precision of the estimation of PMI is higher in cases of death by hanging PMICALC thus represents an improved statistical approach for forensic pathologist using analytic data taken from VH to calculate PMI. It improves this accuracy in PMI estimation when the cause of death is known Faster, more accurate and reliable PMI of a recent death than that given by a simple linear regression Furthermore, PMI estimates are provided in just 20 min [49]										None				
Tandem Mass Spectrometry is an appropriate tool for [Hx] determination. In fact, LOD and LOQ found in this study are smaller if compared with those obtained in other papers Results of imprecision and analytical recovery were satisfactory for all tested compound, except for [UA] The extraction efficiency (higher than 50%) was high. Adequate accuracy and good precision results were obtained for [Hx] As the concentrations of blood contaminant become higher, a decrease in [Hx], [G] and [X] is observed. However, no significant correlation between the 5 levels of blood contamination was found in any of the 15 samples analyzed This method could be useful to identify samples with blood contamination due to improper extraction of the VH. Even at low levels of contamination (when not microscopically evident, and when Hx concentrations are not affected), the two MRM transitions of [UA] disappear [50]										None				
Estimates could be given knowing – at least– values of [Hx] and [K^+^] in VH. The accuracy could be improved if rectal and ambient temperature are considered. Moreover, the application of AM and SVM models after Munoz et al. is recommended Although estimates, circumstances of death and expert opinion will be decisive [68]										None				
Metabolomic analysis of anatomically isolated fluids (AH, VH) is more suitable for PMI estimation than serum A good linear correlation (r > 0,5) is found between PMI and the concentration of following analytes in ocular fluids: creatine, betaine, glutamate, glycine, choline and Hx. Positive, but lower correlation emerged for the following analytes: alanine, inosine, succinate, glycerol. Hx, creatine and glutamate are the only metabolites that show a statistically significant positive trend in all three biological fluids. Furthermore, Hx, choline, creatine, betaine, glutamate, and glycine have the best correlation with PMI The results show that using three metabolites (creatine, choline, and betaine) for AH, and two metabolites (creatine and betaine) for VH, significantly reduces the error in PMI estimation. Including additional metabolites in the regression model does not further improve the accuracy of the estimation Glucose and pyruvate exhibit a negative correlation with PMI, characterized by an exponential decay, making them unsuitable for direct PMI estimation. However, they can be used to confirm PMI measured by other methods: a glucose concentration above 2 mM or pyruvate concentration above 60 µM in AH or VH almost certainly indicates that the PMI is below 24 h For other analytes, no correlation was found [39]										Post-mortem samples were taken from donors which differed by the health status and age before death Causes of death and conditions of the body location before transportation to the morgue were not homogeneous				
Post-mortem modifications of the PF metabolome are correlated with PMI, and this relationship is quantitatively described in a predictive model. No differences in the NMR spectra of proteins and large molecules were observed between samples pre-treated with ultrafiltration or liquid–liquid extraction, with both methods yielding the same prediction error. However, liquid–liquid extraction (LLE) allows for the exclusion of ethanol, which overlaps with 3-hydroxybutyrate Samples with a PMI greater than 100 h exhibit the highest prediction errors, and non-linearity starts to appear between 120 and 160 h after death. In total, 20 metabolites were found to be relevant in the regression analysis. Of these, 11 metabolites were shared between the ultrafiltration and liquid–liquid extraction protocols: choline, ethanolamine, glycine, Hx, formate, and trimethylamine. Notably, Hx showed a decreasing trend [69]										Limited number of samples Not all PMIs were precisely known (some were estimated based on circumstantial evidence/classical thanatological signs) Intervals between 120 and 160 h were not sampled Underrepresentation of samples with PMI > 100 h Most of the corpses were stored cooled in the morgue before autopsy for varying durations The only analytic platform adopted is not appropriate for the lipophilic phase				
Reliable estimates for mean normal [Hx] in VH of living humans without hypothermia is 7,6 µmol/l. [Hx] and [K^+^] slopes become steeper with increasing ambient temperature During the first hours after death, the scatter of vitreous [K^+^] was greater than that of vitreous [Hx] [23]										None				
For VH [Hx] in VH increases linearly with PMI. The linear rise begins immediately after death. Vitreous [K^+^] correlation with PMI is stronger than that of vitreous [Hx]. The conclusion of Rognum et al. about vitreous [K^+^] scatter being greater than [Hx] can’t be confirmed Significant interindividual differences in VH values were observed. If these discrepancies are substantial, later samples may exhibit lower [Hx] values compared to earlier samples For CSF An exponential rise of [Hx] in CSF emerged, with the steepest slope in first 15 h and an immediate rise of [Hx] after death. Equilibration is achieved at 20 h Furthermore, [K^+^] in CSF exhibits an exponential rise over PMI [20]										Influence of hypoxia				
Different mean values of [Hx] in VH emerged among the different groups of cause of death. After application of Mann-Withney U test, no significant difference emerged in [Hx] in CSF among the three groups. Instead, [Hx] in VH showed no significant difference between SIDS cases group and other causes of deaths group, whilst concentration in case of cardiac/pulmonary death were lower [70]										None				
There was no significant difference observed in alcohol levels. Additionally, no discrepancy was noted between eyes or sexes. However, there was a significant difference in the concentrations of [Hx] and [K +] in VH between the hanging and non-hanging groups, with the former showing a higher R^2^ value. Furthermore, the most favorable results were obtained when cases with urea levels below 30 mg/dl were excluded [48]										None				
The higher the temperature, the higher the [Hx] in VH Five models were elaborated by Cordeiro et al. for PMI estimation The increase of [Hx] in VH can be observed up to 120 h after death Age and sex do not have any impact of [Hx] According to Salam et al.'s study, the highest [Hx] and [K +] are typically observed in the 24–60 h post-mortem interval. In this timeframe, estimates of PMI are higher up to 60 h after death [71]										None				
SVM and AM models provide a more accurate prediction of PMI from vitreous [K^+^], [Hx] and [U] than conventional LR models [Hx] increases mainly in the first half of the PMI interval investigated in the study, up to concentrations of 80 µmol/l The SVM model exhibited greater precision than the AM model AM2, when considering cause of death subgroups (hanging or non-hanging), demonstrates greater precision compared to AM without considering cause of death [72]										Subgroup with hanging as cause of death is small (n: 35)				
In human blood samples, [Hx] remains below 1 µmol/l up to 48 h post-mortem. However, beyond this time frame, it notably increases to 18 µmol/l by 96 h, indicating a 30-fold increase. This pattern contrasts with rat blood, where [Hx] remains stable over the same 96-h period [56]										None				
[Hx], [G] and [purine] in cortical bone shows a significant inverse relationship with PMI (in case of PMI < 20 years), following a decreasing exponential model. No statistically significant relationship was found for the group with PMI greater than 20 years [73]										None				
The relationship between T_rec_, [K^+^], and [Hx] with PMI shows a non-linear trend. From an estimation standpoint, Model 3 is considered the optimal choice as it maximizes the R^2^. However, this model does not consider T_amb_, and since T_amb_ is recorded at a specific time, not taking in account its change since death. For a prediction purpose, Model 1 is the most appropriate, as it shows the lowest mean squared error. Age has no influence on PMI estimation. The weight of the corpse has a significant impact on cooling speed, and therefore it should be taken into consideration when estimating the PMI. [Hx] and [K^+^] increase mainly in the first hours after death, but correlation becomes weaker at higher PMIs. None of the following factors showed statistically significant effects in prediction: cause of death, resuscitation maneuvers, age, eye (with no differences between left or right eye), sex, or other analytes [74]										None				
[Hx] and [L-lactic acid] in VH increase with rising ambient temperatures at the time of death. Therefore, PMI prediction is more accurate if the [Hx] is corrected by ambient temperature Correlation between [Hx] in VH and PMI is more precise in winter and less accurate in spring and autumn No significant change was observed in response to little temperature variation in summer and winter. Instead, significant changes were noted in spring and autumn [55]										Information about causes of death and disease were not available				
[Hx] and [uric acid] in VH differ significantly in violent and natural deaths, with higher values observed in case of natural deaths [Hx] values result higher in women than in men. Higher [Hx] and [uric acid] were also found in samples from older subjects A positive correlation between Hx and PMI was noted By dividing samples in two groups based on PMI (first group: 0–12 h; second group: 13–24 h), statistically significant differences between groups for [K^+^] and [Hx] emerged Conducting joint measurements of [Hx], [uric acid], and [K^+^] with a multivariate regression analysis yields improved results in predicting PMI [75]										None				
The elaborated electrochemical biosensor has a LOD lower than the minimum [Hx] observed in VH, demonstrating its practical utility for PMI detection in VH samples. The recovery score further confirms the effectiveness of the electrochemical biosensor for detecting PMI based on vitreous [Hx] [76]										None				
The OMICs method provides classification models incorporating different markers to assess short- and log-term PMIs with high accuracy from human bones samples Short-term PMIs is precisely estimated though metabolites and lipids models, whilst proteomic is applicable for long-term PMIs [54]										Small sample size				
There is no statistical difference for the results obtained by classical methodologies and SIA for detection of [Hx] and [K^+^] By applying SIA for detection of vitreous [Hx], the latter increases linearly with PMI [Hx] in VH samples showed less scatter compared to [K +], indicating that measuring vitreous [Hx] could be highly beneficial for determining PMI. SIA enables two determinations to be conducted within the same sample using a single system and analytical cycle [47]										None				
In the review Ambient temperature has a strong influence on vitreous [Hx], but it’s often omitted in models The increase of vitreous [Hx] and [K^+^] are correlated [Hx] in the first 24 h of death are less dispersed than those of [K^+^], but estimates are less accurate than those obtained combining [K^+^] and [Hx] Many factors influence PMI estimates, such as trauma, eye diseases, hydro-electrolytic disturbance, being the latter often considered in studies. Age is not an influencing factor In most papers a strong correlation in linear regressions is shown The slope of the linear trend appears steeper in the first 6 h Although strong correlations were found out, the elaborated regressions were not applied to an independent dataset, except for Cordeiro et al. study Prolonged hypoxia may induce an increase of [Hx]. Furthermore, alteration in [Hx] could be also induced by alcoholism The most common used technique for VH collection if aspiration thorough a 18-20G needle, followed by centrifugation at 3000 rpm for 10 min There is a great variation in formulas adopted to estimate PMI, due to differences in sample sizes, collection, and pre-treatment, such as analytical technique For the retrospective cohort study For the estimation of PMI, VH biochemistry was adopted in just 22 occasions: 10 used VH biochemistry on its own, three combining it with cadaveric phenomena plus entomology; two combining it with cadaveric phenomena, two combining it with temperature In case of VH biochemistry, the most adopted formula was that of Munoz et al. (n: 8) and that of Madea et al. (n: 4) [57]										In the review The selected retrospective studies show many limitations				
The proposed approach (high-performance liquid chromatography method) has a better R^2^ value, indicating that the error is lower if compared with the traditional method (0.531 vs 0.122) [32]										None				
Heat and enzymatic digestion are not suitable pre-analytical methods for [Hx] determination in VH, as the coefficient of variation is higher compared to other pre-analytical methods (centrifugation and sonication), that have lower variations The lowest range of data dispersion was achieved after centrifugation pre-treatment [21]										None				
There is a linear relationship between PMI and both [K +] and [Hx] in VH. Variations in the equations derived from different studies may be influenced by the methodology used and the composition of the sample group The accuracy of PMI estimation can be enhanced by combining estimates that consider both [K +] and [Hx] [22]										Some estimate result in negative values of PMI due to the biphasic behavior of K^+^ before and after 24 h post-mortem Body temperature was not considered Data collection and profile of samples were not ideal because of the inclusion of non-crystal-clear samples. Furthermore, storage of bodies was not carried out at uniform temperature. In addition, no sample came from a cadaver younger than 18 years Chronic illnesses and urea were not considered in estimates The storage temperature was not homogeneous, as some of samples were stored at higher temperature than 4 °C				

^*^ Samples were taken from both human and animals. In this table, only human samples are reported

**Table 2:** Formulas for estimating the PMI

Authors, Year of publication, and References	Formulas including hypoxanthine	Other analytes and variables’ implication
Rognum T.O., et al 2016 [46]	***Hx* × *0,215* + *T x (−0,467)* + *Hx x T x (−0,005)* + *10,353*** *	K^+^ implicated separately in another regression
Muñoz Barús J.I., et al 2010 [43]	/ (AM, SVM)	U and K^+^ are evaluated with AM and SVM, but no formula is available
Lendoiro E., et al., 2012 [44]	/	Guanine and Xanthine are separately evaluated, but no formula is available
Donaldson A. E., Lamont I.L 2012 [62]	/	Sodium, chloride, potassium, calcium, magnesium, phosphate, lactic acid, urea, creatinine, uric acid, ammonia, catecholamines, and ethanol are studied in the review, but no formula is available
Bande M. F., et al 2023 [63]	/ (AM, SVM)	U and K^+^ are evaluated with AM and SVM, but no formula is available
Zelentsova E.A., et al 2020 [39]	/	*PMI(AH)* = *0.0091* × *[Creatine]* + *0.081* × *[Choline]* + *0.18* × *[Betaine]* + *3.2*
Chigine A., et al 2023 [64]	/ (NMR)	Acetate, betaine, butyrate, choline, citrate, ethanolamine, formate, glycine, inosine, mannose, methanol, myoinositol, nicotinurate, proline, proprionate, β-alanine, trimethylamine, uracil, and uridine are evaluated, but no formula is available
Zięba S., et al 2021 [65]	*PMI* = *0.027* × *Hx* + *15.401* *PMI* = *0.978* × *K* + + *0.014* × *Hx* + *9.178* *PMI* = *0.667* × *K*^+^ + *0.003* × *Hx—0.191 rigidity* + *4.907 hypostasis* + *4.68 corneal turbidity—13.624*	10 formulas including K^+^ are reported in the review. **Of them, one is a multivariate regression including both Hx and K**^**+**^
Rognum T. O., et al 1991 [16]	/	K^+^ is evaluated, but no formula is available
Madea, et al 1993 [13]	/	K^+^ is evaluated, but no formula is available
Carpenter K.H., et al 1993 [66]	/	No
Muñoz Barús J.I., et al 2002 [42]	General regression: ***[HX]* = *26,459* + *3,017PMI*** For hanging group: ***[Hx]* = *15,161* + *4,957PMI*** For non-hanging group: ***[Hx]* = *28,155* + *2,733PMI*** For hanging group if [U] ≥ 30 mg/dl: ***[Hx]* = *17,438* + *4,567PMI*** For non-hanging group if [U] ≥ 30 mg/dl: ***[Hx]* = *30,123* + *2,573PMI***	No
Madea B, Rödrig A 2006 [67]	/	16 formulas including K^+^ are reported in the review
Muñoz Barús J.I., et al 2008 [68]	Linear regression: ***log(PMI)* = *β***_***o***_** + *β***_***1***_***[K***^**+**^***]* + *β***_***2***_***[Hx]* + *β***_***3***_***[U]* + *ε*** Additive Model: ***log(PMI)* = *α***_***o***_** + *f***_***1***_***[(K***^**+**^***)]* + *f***_***2***_***[(Hx)]* + *f***_***3***_***[(U)]* + *ε*** Additive Model 2: $\log\left(PMI\right)\beta_1+\beta_21_{D=h}+f_3\left(\left[U\right]\right)+f_{1h}\left(\left[K^+\right]\right)1_{D=h}+f_{2h}\left(\left[Hx\right]\right)+f_{1h}\left(\left[K^+\right]\right)1_{D=\overline h}+f_{2\overline h}\left(\left[Hx\right]\right)1_{D=\overline h}+\varepsilon$ Support Vector Machine: $\log\left(PMI\right)=f_w\left(\left(\left[K^+\right],\;\left[U\right],\;D\right)^t\right)+\varepsilon=\sum_{i=1}^n\beta_ik\left(\left(\left[K^+\right],\;\left[Hx\right],\;\left[U\right],D\right)^t,\;{\left(\left[K^+\right],\left[Hx\right],\;\left[U\right],\;D\right)}_i\right)+\varepsilon$	[U] and [K^+^] are included with [Hx] in the same models elaborated with LR, AM, AM1 and SVM
Donaldson A.E. and Lamont I.L 2013 [50]	/	No
Pérez-Martìnez C., et al 2017 [69]	For PMI < 20 years: ***y* = *9,007* × *exp (−0,104* × *x)***	Independent formulas are available for guanine and purines (group with PMI < 20 years) and for adenine, guanine, purines, and cytosine (group with PMI ≥ 20 years)
Cordeiro C., et al 2018 [70]	Model 1: ***log (PMI)* = *α* + *f***_***1***_*** (T rectal)* + *f***_***2***_*** (Hx)* + *f***_***3***_*** (K***^**+**^***)* + *f***_***4***_*** (U)* + *f***_***5***_*** (weight)* + *ε*** Model 2: ***log (PMI)* = *α* + *f***_***1***_*** (T rectal)* + *f***_***2***_*** (Hx)* + *f***_***3***_*** (K***^**+**^***)* + *ε*** Model 3: ***log (PMI)* = *α* + *f***_***1***_*** (T rectal)* + *f***_***2***_*** (Tamb)* + *f***_***3***_*** (Hx)* + *f***_***4***_*** (K***^**+**^***)* + *f***_***5***_*** (U)* + *f***_***6***_*** (weight)* + *ε*** Model 4: ***(PMI)* = *α* + *f***_***1***_*** (T rectal)* + *f***_***2***_*** (Tamb)* + *f***_***3***_*** (Hx)* + *f***_***4***_*** (K***^**+**^***)* + *f***_***5***_*** (U)* + *ε*** Model 5: *log (PMI)* = *α* + *f*_*1*_* (T rectal)* + *f*_*2*_* (Tamb)* + *f*_*3*_* (weight)* + *ε*	**Models obtained include K**^**+**^** and U with [Hx] in different combinations**
Go A., et al 2019 [49]	/	Lactic acid is evaluated, but no formula is available
Pérez-Martìnez C., et al 2019 [71]	***PMI* = *4,946* + *0,397* × *K***^**+**^**—*0,110* × *uric acid* + *0,166* × *Hx***	**Models obtained include K**^**+**^** and uric acid with [Hx]**
Liao L., et al 2020 [72]	Rognum et al*.*’s model is applied	
Bonicelli A. et al 2022 [48]	/	Other metabolites (creatine, taurine, hypoxanthine, 3-hydroxybutyrate, creatinine, phenylalanine), such as proteins (histones, hemoglobin, ACTB, VIME) and lipids (3-lysophosphatidylcholine, phosphatidylcholine, phosphatidylinositol) are evaluated, but no formula is available
Passos M.L.C., et al 2009 [41]	***C***_***Hx***_** = *7,12 PMI* + *31,49*** ***PMI* = *0,14C***_***Hx***_*** – 4,42***	Independent formulas for K^+^ (not including Hx) are available *C*_*K*+_ = *0,19 PMI* + *6,23* *PMI* = *5,36C*_*K*+_ *– 33,41. R*^*2*^ = *0,222*
Madea B 2005 [20]	Rognum et al *[Hx]* = *4,2* × *PMI* + *8,6* at 5 °C *[Hx]* = *5,1* × *PMI* + *8,6* at 10 °C *[Hx]* = *6,2* × *PMI* + *8,6* at 15 °C *[Hx]* = *8,8* × *PMI* + *8,6* at 23 °C Madea *et a*l *[Hx]* = *1,29* × *PMI* + *3,69* James et al *[Hx]* = *3,2* × *PMI – 0,15* *PMI* = *0,31* x* [Hx]* + *0,05* Munoz et al *[Hx]* = *3,01* × *PMI* + *26,45* *PMI* = *0,17* x *[Hx]* + *0,17*	14 formulas from nine authors considering K^+^ (not including Hx) are reported in the review
Swann LM., et al 2020 [73]	/	Potassium, amino acids, decomposition products, VOCs, and adipoceres are evaluated in the review, but no formula is available
Li W., et al 2018 [40]	*PMI* = *Hx* × *0,215* + *T x (−0,467)* + *Hx* x* T* x* (−0,005)* + *10,353*	Potassium, sodium, chloride, magnesium, nitrogenous compounds, and amino acids are evaluated in the review, but no formula is available
Peyron P.A., et al 2019 [74]		Potassium, sodium, chloride, calcium, magnesium, phosphate, bicarbonate, carbon dioxide, glucose, pyruvate, inositol, lactic acid, amino acids, ammonia, urea, monoamines, creatine, creatinine, ribonucleotides, uric acid, xanthine, total proteins, enzymes, albumin, tau protein, cardiac troponins, myoglobin, and S-100 protein B are evaluated in the review, but no formula is available
Locci E., et al 2020 [75]	/ (NMR)	Acetate, alanine, ascorbate, BCAA, betaine, carnitine, creatine, creatinine, choline, formate, glucose, glutamate, glutamine, glycerol, glycine, 3-OH-butyrate, histidine, lactate, myoinositol, phenylalanine, pyruvate, succinate, taurine, threonine, and tyrosine are taken in account for their change within NMR spectra at different PMI, but no formula is available
Da Cunha E.C., et al 2022 [51]	Madea et al *PMI* = *3,69* x *[Hx]* + *1,29* Munoz et al *PMI* = *0,172* x *[Hx]* + *0,17* Rognum et al *PMI* = *0,215* x *[Hx] – 0,467* × *T*_*a*_* – 0.05* x *[Hx]* x *T*_*a*_ + *10,35* Cordeiro et al *Log(IMP)* = *α* + *f*_*1*_ x *(t rectal)* + *f*_*3*_ x *(K*^+^*)* + *f*_*4*_ x* (Hx)* + *f*_*5*_ x *(Urea)* + *f*_*6*_ x* (weight)* + *ε* Saldana et al *PMI* = *0,183* x *[Hx]* + *0,133* × *U* + *2,87* x *[K*^+^*]*	23 formulas including K^+^ from 22 authors are reported in the review. Of them, 19 are independent from other analytes, two include Hx and U, one includes U, and one includes albumin Two formulas including U from two different authors are reported in the review. All of them include K^+^ and Hx One formula includes albumin in combination with K^+^
Muñoz JI., et al 2006 [26]	***PMI* = *0,183 [Hx]* + *0,599***	No
Camba A., et al 2014 [14]	/	/
James, R.A., et al 1997 [15]	***PMI* = *0,31* × *Hx* + *0,05***	*PMI* = *4,32* × *K*^+^—*18,35*

^*^Bolding for Hx-related formulas

### Biological matrices and pre-analytical techniques for [Hx]-based PMI estimation

The most frequently studied body tissue was VH, alone in 17 studies, or in combination with CSF in two studies, and with serum and aqueous humor (AH) in one study. One research article studied only the pericardial fluid, one only the blood, and two the bones.

In 13 research papers, centrifugation was utilized during the pre-analytical steps for samples of VH [21, 50, 52, 55, 71, 72, 74, 75], pericardial fluid [69] bone [73], VH plus CSF [70], or VH plus AH plus serum [39]. The centrifugation time was consistent across the research papers, with 10 min being used in 10 studies [21, 34, 48, 55, 69–74]. The temperature, where specified, was always 4 °C [39, 52, 69, 73]. However, the revolutions per minute (rpm) varied, with 300 rpm being the most commonly used [39, 48, 71–73], particularly for VH samples.

Additionally, in 13research papers, samples were stored at low temperatures before analysis [21, 22, 32, 48, 52, 54, 69–73, 75, 76]. In particular, 11 studies reported on the storage of VH, showing significant inhomogeneity in temperatures, ranging from + 4 °C to −80 °C [21, 22, 32, 48, 52, 70–73, 75, 76]. In one study [54], demineralized bone powder was stored at two different temperatures (initially at −80 °C, then at −20 °C).

### Methods for PMI estimation employing Hx concentration

Regarding Hx concentrations in different body biofluids for PMI estimation, nine research papers provided specific formulas (twenty altogether) (Table 2). In particular, four research articles presented multiple regression models, for example, after stratification by cause of death and urea concentration [48], after the adoption of Additive Models (AM) [74] and Support Vector Machines (SVM) [49, 72], after application of different analytes’ combination in the models [72, 74], or interchanging PMI and [Hx] as independent or dependent variables [47]. Furthermore, 10 out of the 20 formulas considered [Hx] as the sole variable for PMI estimation. Instead, in three formulas, the concentrations of K^+^, urea, and Hx were required for PMI evaluation [72, 74]. Additionally, two formulas incorporated the values of the abovementioned three analytes plus a parameter indicating the cause of death, derived through AM and SVM methods [72]. Other regression models, each represented only once in the selected research papers, included the following variables’ combinations: Hx and temperature [52]; Hx, K^+^, urea, weight, and rectal temperature [74]; Hx, K^+^, and rectal temperature [74]; Hx, K^+^, urea, weight, ambient temperature, and rectal temperature [74]; and Hx, K^+^, urea, ambient temperature, and rectal temperature [74]. Some research papers described the relationship between Hx concentration and PMI without explicit formulas. Specifically, five studies [20, 23, 39, 55, 73] reported correlation coefficients between PMI and [Hx] in VH [20, 39, 55, 70], AH [39], blood [39], and bone [73]. In this way, some authors found out several PMI ranges, which could be greater or lesser than 24 h [70] or than 20 years [73]. Additionally, Go et al. [55] corrected correlation factors based on ambient temperature and seasonality.

The increase in VH [Hx] is expressed as an increase rate by Carpenter et al. [70] and Rognum et al. [23]. Specifically, Carpenter et al. [70] reported an increase rate of 8.3 µmol/l/h without temperature stratification. Instead, Rognum et al. [23] calculated the increase rates across different ambient temperature groups, finding the smallest (4.2 µmol/l/h at 5 °C) and the greatest (8.8 µmol/l/h at 23 °C) rates in the lowest and highest temperature groups, respectively. 

### Accuracy of [Hx]-based PMI estimation

The accuracy of PMI estimation from [Hx] is variously expressed in the research articles selected in our review. In particular, R^2^ coefficient, known as the linear coefficient of determination, is the most commonly utilized metric, as it evaluates the effectiveness of a linear regression model in predicting an outcome [32, 47, 48, 69, 71–74]. In studies conducted on VH samples, the estimated R^2^ varies from a minimum of 0,238 [47] to a maximum of 0,831 [72]. Interestingly, higher values are observed in studies recurring to AM and SVM methodologies for PMI estimation [72], in those that propose multivariate regressions [74], or in those that excluded VH samples based on well-defined criteria, such as non-transparent samples [48]. Instead, lower values of R^2^ have been reported when the examiner does not select samples and proposes a univariate formula [47, 73]. The study of Chighine et al*.* [69], conducted on pericardial fluid samples selected by exclusion criteria, resulted in R^2^ values of 0,518. The lowest values of R^2^ were obtained in the studies on bones [73].

Other parameters have been considered in assessing the accuracy of the estimates, such as the mean squared error (MSE) [49, 74], standard deviation (SD) [22, 69, 74], and the root mean squared error (RMAE) [39].

Several authors acknowledged limitations in their research studies [20, 22, 39, 52, 54, 55, 69, 72]. Rognum et al*.* [52] and Chighine et al*.* [69] noted an underrepresentation of PMI in their sample selections. Furthermore, Chighine et al*.* [69] and Bonicelli et al*.* [54] claimed a small sample size. The most commonly declared bias consists of heterogeneity or lack of information about donor characteristics, such as age [22, 52], sex [52], cause of death [39, 55], premortem health status, and illnesses [20, 22, 39, 55, 72]. James et al*.* [22], Zelentsova et al*.* [39], and Chighine et al*.* [69] highlighted that bodies were not stored at uniform temperatures before sampling.

Interestingly, the study reporting the highest number of limitations [22] achieved standard deviation values that suggest a better accuracy than studies not reporting limitations. Indeed, exclusion criteria were applied only to 10 research articles [22, 39, 48, 57, 69, 71, 72, 74, 76]. In general, the sample size is unknown in the studies of Madea and Rödrig [71], and Pérez-Martìnez et al. [73], while it is significantly small in three research studies [47, 54, 68], with fewer than 30 samples analyzed. Moreover, in six cases [21, 32, 39, 47, 50, 56] the cause of death and the donor age were not specified. The explored PMI ranges were not mentioned in seven studies [20–22, 32, 47, 49, 57], or, if mentioned, intervals did not exceed 60 h in seven cases [39, 48, 50, 72–74, 76]. Furthermore, environmental temperature, although known to influence PMI estimates, was considered in only six researches [23, 52, 55, 71, 74, 76].

## Discussion

Most included studies focused on the Hx concentration in the VH. In all cases, an increase of [Hx] during the PMI was observed and a significant linear association was identified between these variables up to 120 h following death [20, 22, 23, 32, 39, 45–50, 52, 54, 55, 57, 70–72, 74–80]. The Hx levels in the VH were also correlated with [K^+^] in the same biological matrix [22, 47, 49, 50, 52], but it was observed that [K^+^]’s variations are more easily affected by the outer temperature [46]. However, post-mortem changes in [Hx] were also found to be affected by the ambient temperature, prompting most PMI estimation models to include outer temperature as a correction factor [36, 39, 52, 68, 74, 78].

Some articles investigated changes of [Hx] in other biological fluids over time, such as CSF [20, 70, 81], pericardial fluid [69], and blood [39, 56, 77]. The behavior of [Hx] in all fluids is similar, with a consistent increase over time after death. However, it was suggested that anatomically isolated compartments grant more reliable estimations for the PMI than blood, as the [Hx] increase in the former is more linear [39]. When CSF samples were obtained, an exponential increase of [Hx] was observed during the first 24 h following death [20, 70, 81].

One study also employed bone matrix samples from long bones, observing a significant decrease of the [Hx] over time in this matrix [73]. Bones and pericardial fluid [69] were the only biological matrices in which a decline in [Hx] concentration was detected.

Many articles focused on the impact of hypoxic death (especially regarding death by hanging) on [Hx] kinetics in various compartments. In all cases, the increase in [Hx] was lower when hanging was the cause of death, due to the reduction in nucleic acid metabolism caused by systemic hypoxia [46, 48, 79]. A similar phenomenon was noted in a study that focused on pre-mortem hypoxia in pediatric autopsies; in this paper, the increase in [Hx] was more significant during the first 24 h post-mortem, after which the rate of increase slowed significantly [70].

For accurately estimating non-prolonged PMIs, VH proved to be the most suitable matrix, as high R^2^ values are reached by implementing multivariate regressions and more flexible methodologies (such as AM and SVM) or applying exclusion criteria to select only high-quality samples. It is consistent with the existing literature, in which VH is considered a stable biological matrix because of its less susceptibility to physical-biochemical conditions (such as bacterial contamination, putrefactive processes, and skull traumas) than other biofluids [20–22, 37–45, 82].

In this context, the extraction of the VH should be performed with a precise technique and by taking care during the extraction procedure to avoid vascular injuries, which might lead to hematic contamination. Indeed, any fluid that is not clear could produce erroneous results and should be discarded [2, 22, 24, 48].

Chighine et al*.* [69] also reported favorable results by proposing pericardial fluid as a matrix for [Hx] detection to estimate PMI. This finding aligns with existing literature in the field of thanatochemistry, which recognizes pericardial fluid – as well as VH—appropriate for post-mortem investigations due to its resistance to autolysis and putrefaction [69]. Nevertheless, pericardial fluid is also influenced by pericardiac and cardiac homeostasis, thus requiring an accurate selection of good-quality samples before BPMs analysis [21, 83].

Linear regression was the main statistical analysis method for PMI estimation, using [Hx] in the investigated body fluid as an independent variable. Other independent variables included the body temperature, the interaction between these two variables expressed as their product, the ambient temperature [21, 46–48, 50, 52, 70–73, 75, 76, 79]. In some cases, computer-based techniques were used by involving more complex regression models such as additive models and support vector machine [49, 72, 74, 84, 85], eventually supported by machine learning systems [86]. Correlation analysis was also frequently employed as a preliminary analysis technique [70].

In other cases, PMI estimation was based not only on VH [Hx], but also on other metabolites’ concentrations quantified in the same matrix, such as K^+^and urea [54, 68, 69]. Additionally, factors such as the cadaver’s weight and the cause of death (hanging vs. non-hanging) were also taken into consideration in the analysis [20, 48, 68, 70, 75].

Various techniques have been employed for quantifying [Hx] in organic fluids. Among them, high performance liquid chromatography (HPLC) and/or capillary electrophoresis are commonly employed when VH serves as the biological matrix [21, 46, 49, 52, 55, 70, 74, 75, 77–79]. The limit of detection (LOD) for vitreous [Hx] was 2.5 µM in a HPLC-based study, in which the limit of quantification (LOQ) was 10 µM; this was deemed to be appropriate for [Hx] determination [50, 75]. A different paper relying on liquid chromatography identified a LOD of 10 µM and a LOQ of 50 µM [55].

Nuclear magnetic resonance was also used for [Hx] quantification, not only in the VH but also in the serum and AH [25, 39, 69, 77, 80]. In other cases, reverse phase column was used to measure [Hx] in the VH [48, 72], as well as spectrophotometric techniques [47] or tandem-mass spectroscopy [55, 68]. When bone tissue was used, mass spectrometry was employed instead [73]. More sophisticated techniques involved the usage of carbon nanofibers as a substrate for electrochemical measurement of [Hx] [76], paving the way for novel, rapid, and cost-effective technologies for metabolite detection [87]. Additionally, results obtained by utilizing sequential-injection analysis (SIA) showed no statistical difference with those of classical methods, suggesting an easy-to-use alternative methodology for PMI estimation [47]. These findings indicate that the research into new methodologies for determining PMI through metabolite quantification could provide new perspectives, enabling increasingly precise estimates of the time since death.

PMI estimates obtained from [Hx] quantification in the VH using linear regression showed a good level of accuracy, correlating well with the observed time after death. SD errors showed a non-linear distribution, with a 95% confidence interval around 2.5 h when [Hx] was around 150 µmol [52]. More accurate results were observed when more complex models were used [46, 49, 72, 74].

In multivariate analysis, SD error in calculation was estimated at 30 h, while SD error in cross-validation was 38 h and SD error in prediction was 33 h; however, cadavers with the highest PMI showed significantly worse estimates of the PMI based on [Hx]. Indeed, the reported error significantly improved (by 13–15 h) when the PMI window was shortened from 170 to 100 h [69].

Hx demonstrated a stronger correlation with PMI than other BPMs [22, 23, 45, 78]. However, other studies indicated that VH [K^+^] may be a more precise tool for PMI estimation than VH [Hx] due to a reduced margin of error [21, 25, 79].

Some papers specifically suggested that [Hx] can be used as a marker of prolonged vital hypoxia in hanging victims, thus being useful for corroborating suspicious hanging cases’ investigations [20, 48, 70, 75, 79]. Interestingly, a paper by Muñoz Barús et al. [48] showed that the best results were obtained after excluding cases with VH [urea] < 30 mg/dl. The use of ambient temperature-based correction was shown to improve the accuracy of PMI estimates [55].

Bone analysis-based PMI estimation was deemed accurate for longer time periods, due to the prolonged persistence of Hx in the bone tissue [54, 73].

The methodological differences between post-mortem biochemistry and metabolomics are critical for understanding the strengths and limitations of each approach. Traditional biochemistry, focusing on individual BPMs, offers a straightforward and well-established method for PMI estimation [22, 23, 32, 47, 52, 70, 76]. However, its univariate approach (typically linear regression-based) provides narrowly focused insights, thus limiting its ability to capture the full complexity of post-mortem biochemical changes. On the other hand, metabolomics provides a more comprehensive perspective by analyzing the collective behavior of numerous metabolites [39, 54, 69]. Its multivariate approach (such as principal component analysis and partial least squares-discriminant analysis) not only enhances the depth of analysis but also potentially increases the accuracy of PMI estimations by capturing the interplay of multiple metabolites. For example, the R^2^ value obtained by Chighine [69] pertains to the entire metabolomic profile, reflecting the proportion of variance explained by the model across all measured metabolites. Unlike traditional biochemistry, in which R^2^ often describes the fit for a single variable, in metabolomics it represents the goodness of fit for the entire dataset, emphasizing the holistic nature of this approach. Moreover, metabolomic approaches allow for investigating a broader PMI range and analyzing different matrices, such as bone [54].

These distinctions underscore the unique contributions of both methods. Traditional biochemistry provides robust and focused insights, while metabolomics offers a broader, more integrative understanding of postmortem changes. By delineating these methodological differences, our study enhances the interpretative clarity and forensic relevance of biochemical and metabolomic data in PMI estimation.Ultimately, the key findings identified in this literature review can be summarized as follows:

- Hx determination is more accurate and suitable for longer PMI estimates than traditional methods, which are effective only within the first 24 h postmortem and are more susceptible to external variables.
- VH is the best biochemical matrix for BPMs assay because of its location in an anatomically isolated compartment and lower susceptibility to outer physical-biochemical conditions. Its reliability is further enhanced by adopting multivariate regression models and more flexible methodologies (such as AM and SVM) or applying exclusion criteria to select only high-quality samples.
- Among the main vitreous BPMs, VH [Hx] is a high-performance analyte on which to perform biochemistry-based PMI estimation because it shows a time-dependent linear increase more regular than other vitreous BPMs (such as K) for a time window of up to 120 h.
- [Hx] shows to be less affected by external temperature than other BPMs (such as K), especially when measured in the VH.
- In multivariate analysis, the highest PMIs showed to be associated with worse [Hx]-based estimates (with SD error less than 15 h for PMIs shorter than 100 h).
- The biological matrix on which [Hx] assay allows exploration of PMIs much wider than VH (over 20 years after death), albeit in the form of a significant [Hx] decrease over time, is the bone.

Biochemical approaches offer the advantage of generating specific parameters and explicit formulas focused on [Hx], directly correlating with PMI. In contrast, metabolomic approaches lack parameters specifically correlating [Hx] with PMI, as the correlations generally reflect the entire metabolome rather than isolating single metabolites. 

## Conclusion

Hx proved to be a valuable marker for PMI estimation. Currently, VH appears to be the most suitablebiological matrix in biochemistry-based PMI estimation, with the most reliable formulas deriving from advanced regression models.

However, the consistent literature demonstrated the need for standardization in pre-analytical procedures (such as sample collection, centrifugation, or freeze storage), as well as the establishment of inclusion/exclusion criteria. This standardization is essential to minimize heterogeneity and enhance results and conclusion validity in any research in the specific field. Furthermore, the variables influencing PMI estimation, such as ambient and rectal temperature, body weight, cause of death, and chronic conditions, should be thoroughly considered to overcome the limitations outlined by several authors and maximize the efficiency of such tools in evaluating time since death.

Research on PMI estimation is moving forward. The evidence from the present review may serve as the basis for future proposals of novel and reliable analytical procedures.

## Data Availability

Not applicable.
